# Endurance Capacity Is Not Correlated with Endothelial Function in Male University Students

**DOI:** 10.1371/journal.pone.0103814

**Published:** 2014-08-07

**Authors:** Yan Wang, Xian-bo Zeng, Feng-juan Yao, Fang Wu, Chen Su, Zhen-guo Fan, Zhu Zhu, Jun Tao, Yi-jun Huang

**Affiliations:** 1 Physical examination center, The First Affiliated Hospital, Sun Yat-Sen University, Guangzhou, China; 2 Education School of Sun Yat-Sen University, Guangzhou, China; 3 Department of Medical Ultrasonics, The First Affiliated Hospital, Sun Yat-Sen University, Guangzhou, China; 4 Department of Hypertension and Vascular Disease, The First Affiliated Hospital, Sun Yat-Sen University, Guangzhou, China; 5 Department of Pharmacology, Zhongshan School of Medicine, Sun Yat-Sen University, Guangzhou, China; The Chinese University of Hong Kong, Hong Kong

## Abstract

**Background:**

Endurance capacity, assessed by 1000-meter (1000 m) run of male university students, is an indicator of cardiovascular fitness in Chinese students physical fitness surveillance. Although cardiovascular fitness is related to endothelial function closely in patients with cardiovascular diseases, it remains unclear whether endurance capacity correlates with endothelial function, especially with circulating endothelial microparticles (EMPs), a new sensitive marker of endothelial dysfunction in young students. The present study aimed to investigate the relationship between endurance capacity and endothelial function in male university students.

**Methods:**

Forty-seven healthy male university students (mean age, 20.1±0.6 years; mean height, 172.4±6.3 cm; and mean weight, 60.0±8.2 kg) were recruited in this study. The measurement procedure of 1000 m run time was followed to Chinese national students Constitutional Health Criterion. Endothelium function was assessed by flow-mediated vasodilation (FMD) in the brachial artery measured by ultrasonic imaging, and the level of circulating EMPs was measured by flow cytometry. Cardiovascular fitness indicator - maximal oxygen uptake (VO2 max) - was also measured on a cycle ergometer using a portable gas analyzer.

**Results:**

1000 m run time was correlated with VO_2_max (r = −0.399, *p*<0.05). However, there were no correlations between VO_2_max and FMD or levels of circulating CD31+/CD42- microparticles. Similarly, no correlations were found between 1000 m run time and FMD, and levels of circulating CD31+/CD42- microparticles in these male university students (*p*>0.05).

**Conclusion:**

The correlations between endurance capacity or cardiovascular fitness and endothelial function were not found in healthy Chinese male university students. These results suggest that endurance capacity may not reflect endothelial function in healthy young adults with well preserved FMD and low level of circulating CD31+/CD42-EMPs.

## Introduction

The Chinese National Survey in 2010 on Students' Constitution and Health revealed a continuous decline of university students' physical endurance over the past 20 years, supported by declining results in 1000-meter (1000 m) run time [1]. Although other countries chose different tests to measure endurance capacity, according to Chinese National Students Constitutional Health Criterion, a 1000 m run of male or a 800 m run of female university students still have been recommended as a measurement of cardiovascular fitness [2]. An increasing number of studies have shown that endurance capacity correlated with cardiovascular fitness in students, healthy subjects, diabetes patients and patients with coronary artery disease [3]–[5]. However, does it suggest how severe such a decline of endurance capacity of Chinese young adults means to their cardiovascular system?

Endothelial dysfunction has been suggested as the initial step to the pathogenesis of cardiovascular disease (CVD) [6], [7]. Recently, a study demonstrated that there's a linear relationship between maximal oxygen uptake (VO_2_max), a good indicator of cardiovascular fitness, and endothelial function measured as flow-meditated vasodilation (FMD) in brachial artery of healthy male adults [8]. Compared with the sedentary elderly, regular physical activities improved the elderly athletes' FMD and VO_2_max [9]. Furthermore, it has been reported that the improvement of endothelial function implicated increase of exercise oxygen uptake during atorvastatin therapy for patients with post myocardial infarction [10]. Based on these studies and the correlation between endurance capacity and cardiovascular fitness, it is reasonable to hypothesize that there may be a relationship between endurance capacity and the endothelial function in university students.

Recently, it has been reported that alteration of circulating EMPs, mostly defined as CD31+/CD42- microparticles, emerged as a new marker for endothelial injury in response to various stimuli [11], [12]. Even though measurement of flow-meditated vasodilation (FMD) in brachial artery has been regarded as the common method to assess endothelial function, it was demonstrated that circulating EMPs was increased in offspring of patients with coronary artery disease, whose FMD were in normal range [13]. This suggests EMPs might be an earlier and more sensitive marker of endothelial dysfunction in apparently healthy subjects even if FMD still remains normal. Accumulated evidence indicates that increased EMPs per se actively contribute to deterioration of endothelial homeostasis partially via increasing oxidative stress in endothelial cells [14], [15].

Regular aerobic exercise improves physical fitness, especially improve endurance capacity. And it was demonstrated that acute endurance exercise or long-term aerobic exercise lowered the levels of circulating EMPs [16]–[18]. However, it is still unknown whether basic endurance capacity will correlate with circulating EMPs and reflect the status of endothelial function. It is also unclear whether endurance capacity can be used as a predictive measurement of cardiovascular disease in young adults. Therefore, the purpose of this study was to assess the relationship between endurance capacity and endothelial function in Chinese university students.

## Methods

### Subjects

This cross-sectional study was approved by the Ethics Committee of Zhongshan School of Medicine of Sun Yat-Sen University. Each subject was aware of the investigative nature of the study and gave written informed consent. Forty-seven apparently healthy male student subjects were recruited from grade 2 of the Zhongshan School of Medicine, Sun Yat-Sen University. We recruited subjects with relatively stable endurance capacity, which means 1000 m run time in three consecutive semesters of those subjects, including the latest test two weeks before the lab tests were relatively stable (ranged between mean run time ± 10%). Subjects with clinical history of cardiovascular diseases such as hypertension, hyperlipidemia, diabetes mellitus, ischemic heart diseases, and acute pathologies were excluded. Subjects with familial history of cardiovascular disease were also excluded. All subjects declared no smoking, no alcohol and no medicines at least two weeks before the study.

### Analyses

PE-conjugated monoclonal antibody against PECAM-1(PE-CD31) and FITC-conjugated monoclonal antibody against Platelet Glycoprotein Ib (FITC-CD42b) were used to identify EMPs. All the flow cytometry labeling reagents including PE- and FITC-conjugated monoclonal IgGs were obtained from Immunotech (France). Flowcount fluorospherical beads used to determine microparticles' absolute values and 1-µm calibrant beads used to define the upper size limit of the microparticles were from Beckman Coulter (USA).

### Procedures of study

The 1000 m run time was measured on the running track in a stadium of Zhongshan School of Medicine. Following the procedures of Chinese National Students Constitutional Health Criterion, the test was conducted from 16∶30 to 17∶30 at an outdoor temperature of 24°C −27°C. Furthermore,all subjects were clearly instructed to maintain their lifestyle, especially keep their physical exercise habits during the whole study.

The subjects refrained from strenuous physical exercise and consuming caffeine for at least 24 hours before cardiopulmonary test and other measurements. Two weeks after the last 1000 m run test, the venous blood sample for circulating EMPs and routine laboratory tests were drawn from the forearm at early morning in fasting status. After at least 15 minutes resting in a supine position, FMD test was carried out in a controlled environment maintained at 23°C. Then in a laboratory maintained at 25°C∼27°C and relatively humidity at 50%∼70%, the exercise cardiopulmonary test was performed 2∼3 hours after meal to measure VO_2_max, ventilation volume (VE) and heart rate (HR). Routine laboratory tests including serum total cholesterol, total triglyceride, low density lipoprotein-cholesterol, high density lipoprotein-cholesterol and fasting plasma glucose were measured by standard laboratory methods.

### Circulating CD31+/CD42- microparticles isolation, immunolabeling and flow cytometric analysis

**Figure 1:**
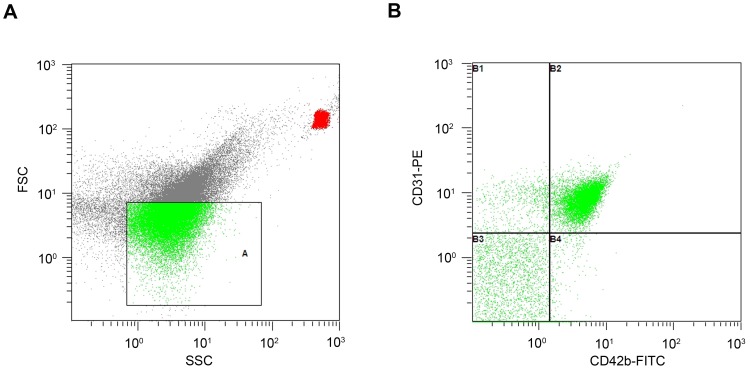
Representative graphs of flow cytometry analysis of circulating microparticles from one of the subjects. A. Circulating microparticles are represented on a forward scatter/side scatter dot plot. Total microparticles are defined as events with a size of 0.1 to 1.0 µm gated in window A using 1 µm-diameter calibrant beads as interior criterion prior to the sample testing. B. Size-selected events are plotted according to their fluorescence for specific CD42b-FITC(PMT2) and CD31-PE(PMT3) binding on a two-fluorescence plot. Events included in B1 section (CD31+/CD42b-) were considered microparticles mainly of endothelial origin.

Circulating CD31+/CD42- microparticles were isolated from 4 ml of venous citrated blood drawn from forearm as described in our previous reports [11]. Briefly, the platelet-free plasma was separated from the 4 ml blood sample by centrifugation at 160×g (10 min) then 1000×g (6 min). Fifty microliters of the platelet-free plasma was incubated with anti-CD31-PE (5 µl/test) and anti-CD42b-FITC (5 µl/test) antibodies or their respective isotypic immunoglobulins at room temperature for 30 min in the dark with regular shaking. Samples were analyzed on an EPICS Altra (Beckman Coulter, USA) flow cytometer by an independent operator who was unaware of the subject status. After using isotypic control to exclude non-specific fluorescence, events with a 0.1 to 1 µm diameter calibrated by 1 µm calibrant beads were identified in forward scatter and side scatter intensity dot plot, gated as microparticles (Fig. 1A), and then analyzed on a two-fluorescence plot. CD31+/CD42- microparticles were defined as events that had a size <1 µm, positively labeled by anti-CD31-PE and negatively labeled by anti-CD42b-FITC (CD31+/CD42b- microparticles), that is B1 section in the plot (Fig. 1B). The absolute count of circulating CD31+/CD42- microparticles was determined using the flow-count fluorospheres with known concentration provided by manufacturer, which was added (50 µl/test) into the sample right before flow cytometric analysis. The final concentration of circulating CD31+/CD42- microparticles was expressed as microparticles per µl.

### Measurement of flow-mediated vasodilation (FMD) in the brachial artery

FMD measurement in the brachial artery was performed with subjects in the supine position for the evaluation of endothelial function. All FMD imaging tests were performed by a highly skilled sonographer who was unaware of the study design. As we described previously [19], [20], brachial artery diameter was imaged with a 5–12 MHz linear array transducer ultrasound system at a location 3 to 7 cm above the right elbow. The brachial artery diameters at baseline (D0) and after reactive hyperemia (D1) were recorded. The flow-mediated vasodilation [(D1-D0)/D0×100%] was used as a measurement of endothelium-dependent vasodilation. The repeatability coefficients of flow-mediated vasodilation on the same person in 2-d interval were 0.93.

### Measurement of maximal oxygen uptake

The measurement of VO_2_max was performed by a light-weight breath-by-breath portable gas analyzer (K4b^2^, COSMED, Rome, Italy) and progressive maximal cycle ergometer (Monark 839E, Vansbro, Sweden). Validity and reliability of the K4b^2^ portable gas analyzer have been reported elsewhere [21]. All subjects performed an incremental graded exercise on the cycle ergometer by starting with 60 W and an increase of 15 W every minute while subjects were maintaining a pedaling rate of 60 rpm until volitional exhaustion despite verbal encouragement. Subjects met at least 2 of the following criteria at exhaustion: respiratory quotient (RQ)  =  carbon dioxide production (VCO_2_)/oxygen consumption (VO_2_) >1.1, heart rate (HR) >90% of the age-predicted maximum, and a leveling off of VO_2_ despite the increment in the workload. Respiratory frequency, HR, VE, VO_2_, and VCO_2_ were monitored continuously at all time during the exercise cardiopulmonary test by telemetry. VO_2_max corresponded to the plateau (average of 30s) in oxygen consumption, despite the increment in the workload.

### Statistical analysis

Data of this study are expressed as mean±SD except for circulating CD31+/CD42- microparticles as mean±SEM. Statistical analysis was performed with SPSS 13.0 software for Windows (SPSS Software, Chicago, IL). Pearson's correlation test was used to test the associations among variables in the whole subjects. A two-tailed test (P <0.05) was considered significant.

## Results

### 1000m run time correlated with VO_2_max and other indicators of exercise cardiopulmonary measurement

**Figure 2:**
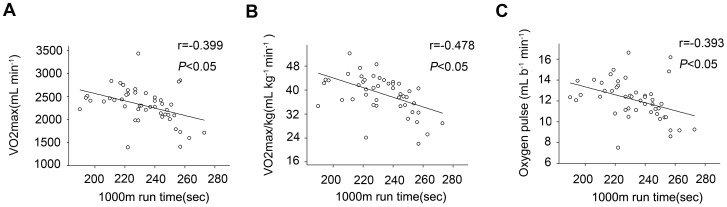
Significant correlations between 1000 m run time and indicators of exercise cardiopulmonary measurement. 1000_2_max (A), VO_2_max/kg (B) and Oxygen pulse (C).

The physiological characteristics of the recruited healthy subjects were shown in Table 1. Figure 1 showed representative graphs of flow cytometry analysis of circulating endothelial microparticles in platelet-free plasma from one of the subjects. As Figure 2 showed 1000m run time significantly and inversely correlated with VO_2_max (r = −0.399, *p*<0.05) and VO_2_max/kg (r = −0.478, *p*<0.05). And there was significant correlation between 1000 m run time and oxygen pulse (r = −0.393, *p*<0.05)

**Table 1:** Physiological characteristics of the subjects (N = 47).

	Mean±SD	Range
Age(years)	20.1±0.6	19–21
Height(cm)	172.4±6.3	161–188
Weight(kg)	60.0±8.2	48.7–96.4
BMI(kg/m^2^)	20.1±2.1	15.9–27.1
SBP(mmHg)	125.5±10.9	107–139
DBP(mmHg)	70.3±10.5	52–88
TC(mmol/L)	4.0±0.7	2.3-5.7
TG(mmol/L)	0.9±0.3	0.4–2.2
LDL-C(mmol/L)	2.44±0.62	1.21–4.11
HDL-C(mmol/L)	1.49±0.29	1.00–2.35
FPG(mmol/L)	4.8±0.2	4.4–5.4
UA(µmol/L)	381±77	266–617
1000 m run time(sec)	232±19	190–273
VE(L)	95.9±20.1	57.1–146.6
HRmax(beat min^−1^)	190±11	164–212
VO_2_max(mL min^−1^)	2336.5±396.3	1395.2–3430.4
VO_2_max/kg(mL kg^−1^ min^−1^)	38.8±6.5	22.0–52.2
Oxygen pulse(mL b^−1^ min^−1^)	12.1±1.9	7.5–16.6
FMD(%)	14.1±3.3	8.8–23.3
Total MPs(events/µl)	1763.4±140.5	618–4421
CD31+/CD42- MPs (events/µl)	83.2±6.3	26.5–229

Values are expressed as Mean±SD, except total MPs and CD31+/CD42- MPs are expressed as mean±SEM. BMI, body mass index; SBP, systolic blood pressure; DBP, diastolic blood pressure; TC, total cholesterol; TG, triglyceride; HDL-C, high density lipoprotein cholesterol; LDL-C, low density lipoprotein cholesterol; FPG, fasting plasma glucose; UA, uric acid; VE, ventilation volume; HRmax, maximal heart rate; VO_2_max, maximal oxygen uptake; FMD, flow-mediated vasodilation; Total MPs, total microparticles; CD31+/CD42- MPs, CD31+/CD42- microparticles.

### VO_2_max was irrelevant to indicators of endothelial function, whether it was FMD or levels of circulating EMPs

There was no correlation between VO_2_max and FMD (r = 0.012, *p*>0.05). Coincidently VO_2_max was not correlated with levels of circulating CD31+/CD42- microparticles (r = 0.169, *p*>0.05) in the all subjects.

### 1000 m run time was not correlated with indicators of endothelial function

**Figure 3:**
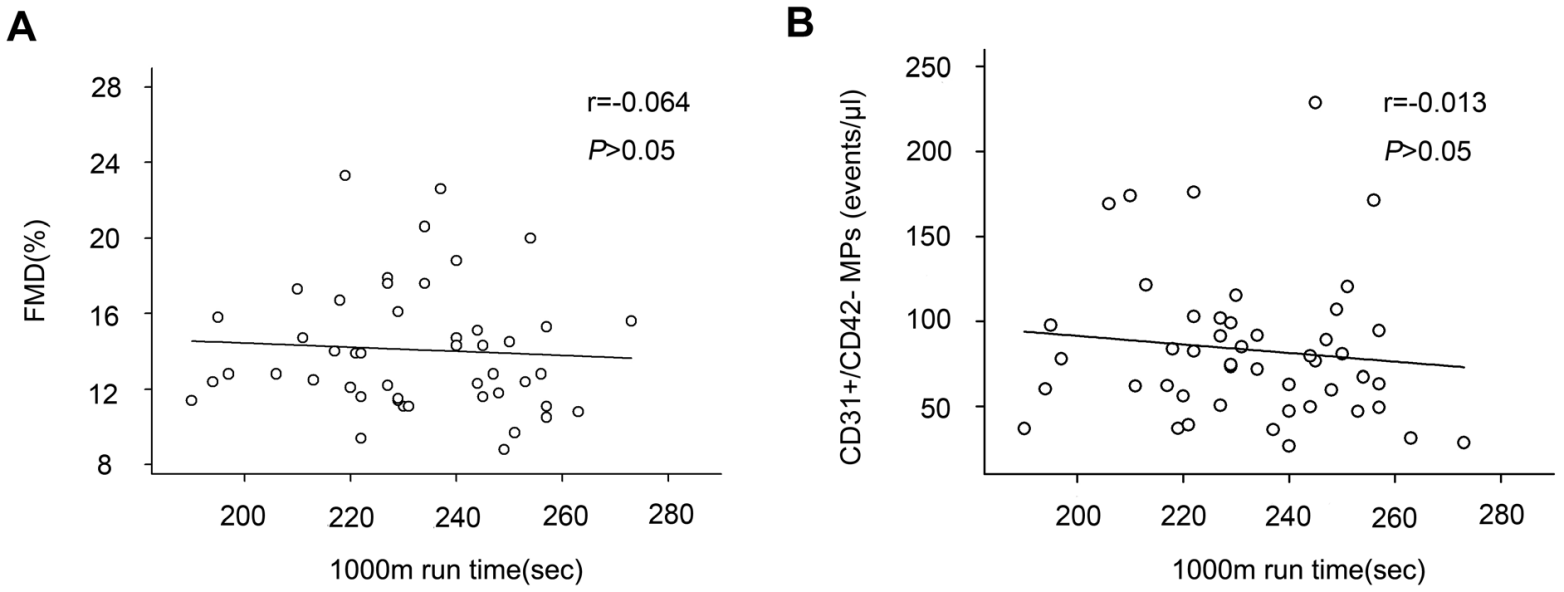
No correlations between 1000 m run time and indicators of endothelial function. 1000(A) nor levels of circulating CD31+/CD42- microparticles (B) in the whole subjects.

As Figure 3A showed that there was no correlation between 1000 m run time and FMD (r = −0.064, *p*>0.05). Neither 1000 m run time correlated with levels of circulating CD31+/CD42- microparticles in the all subjects (r = −0.013, *p*>0.05, Figure 3B).

## Discussion

The major findings of the present study are: 1) VO_2_max was correlated with neither FMD nor the level of circulating EMPs; 2) endurance run time was unrelated to FMD or the level of circulating EMPs in apparently healthy male university students; 3) endurance run time was significantly correlated with VO_2_max. The present study is to investigate the relationship between endurance capacity and endothelial function in male university students. It was demonstrated that FMD was in normal range and the level of circulating EMPs was low in the subjects of the study, and there was no relationship between endurance capacity and endothelial function, suggesting that basic endurance capacity can't reflect endothelial function superficially in male university students, hence basic endurance capacity might not serve as a useful predictive marker of cardiovascular disease in apparently healthy young adults.

Endothelial dysfunction plays an important role in the pathogenesis of cardiovascular disease (CVD) [6], [7]. Previous studies reported that physical training, in particular endurance exercise improved endothelial function, especially via increasing nitric oxide production and bioavailability [22], [23]. Increased nitric oxide production leads to vasodilation and a parallel increase in the blood flow and muscular oxygen delivery, which could invert into better cardiovascular function. Guazzi reported atorvastatin therapy for 3 months in patients with post-myocardial infarction improved FMD in brachial artery and VO_2_max. The authors reckoned the improvement of endothelial function implicated the beneficial therapeutic property of statin in increment of VO_2_max [10]. It has been demonstrated that FMD correlated with VO_2_max in healthy adults and the elderly [8], [9], and the value of FMD of the older athletes was comparable with that of the sedentary young group. These results suggested that keeping endothelial function in steady status should benefit well-conditioning cardiovascular fitness.

However, in our study we didn't find the relationship between FMD in brachial artery and VO_2_max. The possible reason was the study subjects, who were male university students in the same grade, had the similar FMD in normal range. We assumed that FMD of the study population in their university age period might be mainly determined by their original physical status such as heredity background, and physical training might affect FMD mildly in individuals in this age. Actually, our results were in agreement with Ragnom's and Moe's studies [24], [25]. They reported that highly endurance-trained young men or young women and their healthy sedentary control subjects had different VO_2_max but similar FMD, when compared with baseline levels or after a single bout of acute exercise. The authors considered higher cardiovascular function status due to long-term aerobic training didn't improve endothelial function any further in young healthy subjects with well preserved endothelial function. However, in those studies which showed the positive relationship between FMD and VO_2_max, different age groups or subjects within big age span had been compared. Age heterogeneity of the subjects might be the reason why positive results were found in these studies [8], [9]. In fact, Franzoni et al reported FMD correlated with age inversely and correlated with VO_2_max coincidently.

Although the more meticulous experimental parameter-VO_2_max is the reference indicator of cardiovascular fitness [26], 1000 m endurance run performance is easily obtained and widely used to represent cardiovascular fitness in Chinese National Survey on Students' Constitution and Health [1]. In this cross-sectional study we confirmed the relationship between endurance run time and VO_2_max, which suggested basic endurance capacity represented cardiovascular fitness in our subjects. Considering the possible relationship between VO_2_max and FMD, it's possible to excavate a more convenient surrogate measurement to assess endothelial function in young adults. Furthermore, does continuous decline of 1000 m run time suggest endothelial dysfunction in university students? Other more sensitive markers except FMD should be introduced to the study to assess the potential endothelial dysfunction.

The circulating microparticles of endothelial origin are considered as a sensitive marker for endothelial injury [27]. Changes in their plasma levels may provide important clinical information for healthy subjects and patients with endothelial dysfunction or injury [11], [28], [29]. EMPs have also been demonstrated to directly facilitate occurrence of endothelial dysfunction, which is at least partially related to oxidative stress [30]. Our previous study showed that berberine improved endothelial function by reducing EMPs-mediated oxidative stress in apparently healthy individuals [20]. It has been reported that exercise reduced the level of circulating EMPs as a new mechanism of protecting endothelial function. A single bout of prior endurance activity partially prevented the increase of circulating EMPs after a fat meal [16], [17], in parallel with the reduction of reactive oxygen species production in circulating CD31+ cells. But Sossdorf reported the increment of the circulating microparticles delayed to return to the baseline level after 2 hours of the exercise in sedentary individuals, compared with those trained controls, which might contribute to an exercise-induced increase of hemostatic potential [31]. Considering many cities have been holding marathon matches nowadays and sometimes there were sudden deaths happened in apparently healthy young men, loss of endothelial homeostasis and potential procoagulant status might be the possible reason of the events [32]. It's crucial to pay attention to the endothelial homeostasis including levels of EMPs in young university students with continuous declining endurance capacity.

Taken by surprise, relationship between endurance run time and levels of circulating EMPs in male university students had not been demonstrated in this study. It has been reported that the levels of circulating CD62E+EMPs in Africa Americans with inflammatory endothelial dysfunction was significantly decreased after 6 months aerobic endurance physical training [18]. However, a recent study by Boyle et al demonstrated reduced 5 days physical activity didn't increase the level of circulating CD62E+EMPs in apparently healthy subjects [33]. Instead, the level of circulating CD31+/CD42-EMPs was increased after reducing physical activity. Considering the CD31+/CD42-EMPs and CD62E+EMPs populations were respectively thought to be markers of endothelial apoptosis and activation [34], it's more reasonable to measure CD31+/CD42-EMPs in apparently healthy students without substantial inflammation. In this cross-sectional study, it's aimed to excavate the relationship between basic endurance capacity mainly determined by individual heredity background and personal exercise habit,and circulating EMPs. Under these circumstances, no correlation was found between each other. Whereas in those interventional studies focusing on the effects of manipulated exercise on endothelial function and circulating EMPs, most of these studies claimed positive results [16]–[18]. It could be prudently concluded that basic endurance capacity might not reflect endothelial function in individuals of university age period, but long-term follow-up of individuals with different exercise habits is still necessary to monitor whether the progress of endothelial dysfunction will be different among populations with diverse endurance capacity supported by long-term exercise.

It should be pointed out that there were some limitations in the studies. First, this was a cross-sectional study, which suggestive correlations and not casual relationships were observed. Hence, it's impossible to conclude whether declining endurance capacity affected endothelial function or not. Second, the subjects in the current study were in their university age period, whether our results could be extrapolated to other populations with different characteristics remains to be studied. Third, this study selected a narrow sample of subjects, and it just observed the relationship between endurance capacity and endothelial function in the baseline physical condition. Whether there will be any change after an acute exercise or long-term exercise has not been investigated. Further studies with large sample or subjects with different exercise background should be designed to answer these questions.

In summary, this study doesn't demonstrate correlation between endurance capacity and endothelial function, which suggests that endurance capacity might not superficially reflect endothelial function in apparently healthy male university students. The result also implies the disability of endurance capacity for predicting cardiovascular disease in young people with well-preserved FMD and low level of circulating CD31+/CD42-EMPs. Further studies are necessary to follow up changes of endurance capacity and level of circulating EMPs in this study population with aging and investigate the relationship between each other in different pathological conditions.
